# Patients' Contributions to Hospitals

**Published:** 1888-05-26

**Authors:** W. Burdett-Coutts


### PATIENTS' CONTRIBUTIONS TO HOSPITALS.

By Mr. W. Burdett-Coutts, M.P.

*(Continued from p. 113.)*

### United States.

In considering the rise of the Pay System in America, it is to be remembered that before the establishment of the Republic, no single American hospital had an endowment of any kind. Public subscriptions were found to be insufficient for their maintenance, and the American has no sympathy with pauperism. Pay wards, sometimes forming a separate wing, were therefore established, and an income thus obtained which, with that derived from voluntary sources, sufficed to maintain the hospital free from debt; and at the present time from fifteen to forty per cent. of the total cost of maintenance of the older hospitals is usually derived from the patients' payments. Even the large pauper hospitals—for instance, the Belle Vue, New York—occasionally admit pay patients, and possess what they term a pay list. The more recently-established hospitals are now appreciably endowed; but they have preserved, and even more perfectly arranged, the system of pay wards and pay patients. They all now make provision for pay beds, some of these being placed side by side with those occupied by non-paying patients, All receive the same medical attention, nursing and diet; but free patients are required to assist, when able, in nursing, in cleansing the wards, and in other ways. A certain number of beds are also maintained by governors who pay a fixed sum annually for that purpose. Each year an account is drawn up of the exact expenditure upon free and upon paid beds; and, should the expenditure have exceeded the income to any great extent, such a reduction is made in the number of free beds as will re-establish a satisfactory balance between income and expenditure. The Pay System is popular with the medical profession, with social economists, and with the people themselves, and the tendency is more and more to increase the accommodation provided for pay patients, because it is felt that it will prevent many classes from becoming paupers, and will at the same time produce among all classes a wholesome regard for the preservation of their health. But until the medical staff of the hospitals are paid, managers will not be able to claim that the paying wards enable them to make their institutions self-supporting. At the Belle Vue Hospital a patient may pay three guineas a-month—a sum which is about equal to the cost of his maintenance—but the Massachussets Hospital, Boston, is perhaps the best representative establishment in America. The management is vested in the resident physician, under the supervision of the visiting committee. This body, which reserves to itself the right of refusing admission, regulates the various grades of payment, and receives security for the payment of charges of treatment. They may at any time consider the circumstances of applicants before them, and can dismiss any patient improperly admitted, or alter the conditions upon which he has been received. The charge which any patient is to pay is fixed by the resident physician, in accordance with the regulations. The scale of payment varies from under £1 to £7, or even £14 per week; and, in the year 1877, of a total number of admissions of 1,847, 358 paid full fees, forty-eight part fees, and 1,441 were free cases. The average residence of paying patients was 2·84 weeks, and that of free patients 5·54 weeks. Of the male patients, fifty-two were mechanics, forty-three labourers, thirty-one farmers, thirteen children, eighteen seamen, twenty-two clerks, two teamsters, seven domestic servants, two lawyers, three clergymen, three physicians, thirty-one merchants, and seventeen belonging to other professions. Of the females, seven were domestics, twelve children, forty-three wives of well-to-do citizens, seven widows, six spinsters, and clerks, nurses and teachers. In 1876, patients' payments amounted to £2,638; their cost was £2,363, and there was thus a net profit of £275. In the following year this profit was increased to £681. Of the number treated in the free wards the majority were foreigners, and it should also be mentioned that in the out-department no medicine, but only the prescription is given, except in a few exceptional cases.

### Canada, The Cape, etc.

The American system has naturally been largely adopted in the Dominion. At Montreal there is a pay-patient wing, having been erected in connexion with the Western Hospital in the city, possessing the main features of the St. Thomas's Home. The charge for a bedroom and sitting room *en suite* is £1 per day; for a single room, 12*s.*, 10*s.*, 8*s.*, and down to 5*s.*, this payment being inclusive. This pay wing has been erected with the object of providing additional income to enable the managers to secure extra accommodation for free patients who may be dependent upon gratuitous medical relief. It is estimated that the charges made to the pay patients are based on a scale amply sufficient to render it possible for the authorities to defray the cost of one free and one pay patient out of the payments made by each pay patient in a private ward. Similar accommodation is to be found in Australia, French Guiana, at Madras, Buenos Ayres, and the Cape, there being four hospitals at Kimberley, viz., the Upper Class, the General, the Half-Caste and the Kaffir Hospitals. Payment made at the first is 7*s.* 6*d.*, per diem, of the second 4*s.* 6*d.*, and at the two last only a small payment; but should there be any deficiency in the income of these two institutions it is defrayed by the Colonial Government.

### Pay System at Home.

I now proceed to give an account of the Pay System in the many limited and scattered forms in which it exists in this country. It will, of course, be understood that I can only give the results of the answers I have received to my interrogatories, which already amount to more than three hundred reply papers. At the moment of going to press they are still coming in intermittently,

I take, first, the case of the general hospitals of London, not only as the most important in England, but as the hospitals which present, in spite of their great endowments, the greatest necessity for, and, in spite of their admirable management, the greatest difficulties in the way of, an adoption of the Pay System. It is a coincidence of which I gratefully avail myself, that the noble institution, whose hospitality we acknowledge to-night, should at the same time afford us the most eminent example to be found in London of at least one important part of the Pay System. The pay ward in St. Thomas's Hospital, known as St. Thomas's Home, has now been established three years. During that time its receipts (for three complete years) have been £15,999 14*s.* 6*d.*, its expenses £14,073 6*s.* 10*d.*, leaving a credit balance of £1,926 6*s.* 10*d.*, a remarkably satisfactory result from a first attempt. It must be noted that the expenses include three-fourths of the preliminary expenses, and on that point I should like to observe that the preliminary or installation expenses are, not only in this, but in every department of hospitals, a legitimate object of charity. I am sure that if the advantages of this system were fully realized, there would be plenty of contributions from generous and wise people to pay for its installation. I have called St. Thomas's Home an important part of the Pay System. It is not the whole system, for it is exclusive. The patients pay 8*s.* or 9*s.* a day, and for a separate room £1 1*s.* a day, and it is stated that "It has been a very great convenience to clergymen, officers of the Army and Navy, clerks, etc." But between these classes or any class that can pay such sums and the class that can pay nothing, it is obvious that there must be a large range of patients, varying in circumstances and means and necessities, who, in corresponding degree, could and would pay varying proportions of the cost of their treatment, but who, at the present time, rely entirely upon the charity of others. The system as here practised is one which I would desire to see at every great hospital in London, as well for the credit and status of the hospital as for the benefit and more efficient treatment of well-to-do patients. It has many obvious advantages, but that which is most pertinent to the object of this paper is that it makes an automatic contribution of a very considerable sum to the funds of the hospital. But still it is not what I may call the complete Pay System, and those hospitals which by their situation or for any other reason were unable to have such an element, would not be bettered in their resourses by its existing elsewhere. St. Thomas's Hospital has endeavoured to adopt some form of the Pay System in its general wards, by taking a certain number of poor paying patients at 3*s.* per day, but the average number per annum has been only thirty, and the total average payment £130.

ARE GRADUATED WARDS NECESSARY TO PAY SYSTEM?

I must here remark that it is necessary to any complete form of the Pay System that it should run through the general wards. It would be reasonable in practice (although I do not like the idea) to separate off altogether the absolutely non-paying patients; but between them and the class at the other end, viz., patients who can pay the whole cost of their treatment, there would be too many variations to permit of any graduated subdivision of wards. Failing any other method of adjusting the payment to the means of the patient, but only in that case, it might be advantageous to make some sort of classification of the wards, according to the number of beds or extra comforts, admission of friends, etc., so that each patient, by his own desire or that of his relations and friends, would secure for himself the best accommodation he or they could afford. I confess the idea is distasteful to me. I should prefer that in Nature's great republic of disease and suffering, the inmates of hospitals should in the main be possessed of equal rights, while, as in the political State, they should contribute to its income according to their varying positions and means. On the other hand, comparison probes this theory; for in everything else affecting human life, in food, clothing, houses, and all other necessities, comforts, or luxuries, the supply is proportioned to the means; and we have to face the merciless question: Can we support a system in one great department of human requirement which violates this economic law?

(*To be continued*).

